# The Expression of Glycoprotein Genes in the Inflammatory Process of Kawasaki Disease

**DOI:** 10.3389/fped.2020.592122

**Published:** 2020-12-03

**Authors:** Kuang-Che Kuo, Ya-Ling Yang, Mao-Hung Lo, Xin-Yuan Cai, Ho-Chang Kuo, Ying-Hsien Huang

**Affiliations:** ^1^Department of Pediatrics, Kaohsiung Chang Gung Memorial Hospital and Chang Gung University College of Medicine, Kaohsiung, Taiwan; ^2^Department of Anesthesiology, Kaohsiung Chang Gung Memorial Hospital and Chang Gung University College of Medicine, Kaohsiung, Taiwan; ^3^Kawasaki Disease Center, Kaohsiung Chang Gung Memorial Hospital, Kaohsiung, Taiwan

**Keywords:** Kawasaki disease, glycoprotein gene, inflammasome, IVIG resistance, coronary arterial lesions

## Abstract

**Background:** Kawasaki disease (KD) is the most common form of febrile coronary vasculitis disease to occur in children. Early diagnosis and proper therapy can prevent the complication of coronary artery lesions (CAL). The main pathogenesis of KD is an inflammatory process related to the host's genetic characteristics. In innate human immunity, the interaction of leukocytes and glycoprotein plays an important role against microbes. The purpose of our study was to understand the role of leukocytes' glycoprotein genes during the acute phase of KD.

**Materials and Methods:** We enrolled a total of 97 subjects from a medical center. Of those, 24 subjects were healthy controls, and 24 subjects were fever controls; the other 49 subjects were KD patients who had had blood samples taken both before and after IVIG treatment. We collected the total RNA from leukocytes and performed a quantitative polymerase chain reaction for the HP, GRP84, and CLEC4D genes in real time.

**Results:** Compared with both the healthy and fever controls, the upregulation of HP, GRP84, and CLEC4D genes was significant in peripheral leukocytes during acute-phase KD. The transcriptional level of these respective genes not only demonstrated a positive correlation with each other, but were also effective predictors for KD (all auROC >0.87) according to the ROC curve analysis. The hyper-expression of these three genes was significantly associated with IVIG resistance, but not CAL formation.

**Conclusions:** Our study demonstrates that the expression of HP, GRP84, and CLEC4D genes of leukocytes play an important role in the pathogenesis and primary IVIG response during the acute inflammatory process of KD.

## Introduction

Kawasaki disease (KD) is a form of inflammatory disease associated with both genetic and infectious factors. Prior research has found neutrophil activation, reactive serum cytokines, and enhanced formation of neutrophil extracellular traps within the inflammatory process of KD (1, 2). Previous studies have indicated that the regulation of TLRs participate in the pathogenesis of KD by stimulating neutrophil migration (2–4). Other studies have also revealed that both neutrophil migration and transformation were associated with refractory response to IVIG and coronary artery lesions (CAL) in KD (5–7). The chronic sequelae of coronary artery injuries, such as aneurisms, represent a clinical problem that needs to be resolved within the acute stage of KD. Intravenous immunoglobulin (IVIG) can induce neutrophil apoptosis and reduce the subsequent production of active oxygen, thus making it an effective agent for the current treatment of KD (8–10). Therefore, the refractory response (or IVIG resistance) of KD patients has a higher association with CAL formation.

In our previous study, in which we integrated genome-wide DNA methylation with Illumina HumanMethylation450 Bead-Chip and whole gene expression with Human Transcriptome Array 2.0 between KD and controls, we found that the top gene ontology items were also involved in neutrophil migration and chemotaxis (11, 12). We first observed DNA hypomethylation and increased CD177 transcripts in KD compared to the control subjects. Furthermore, CD177 was found to be related to the typical presentation of KD, as well as associated with IVIG resistance in KD patients (11). Moreover, we demonstrated that only the S100A gene family played an important role in the pathogenesis of KD and indicated that a recombinant protein of S100A could enhance neutrophil transendothelial migration (12). Both the function of CD177+ and S100A family can enhance antimicrobial activity, which is associated with the increased production of neutrophil extracellular traps, reactive oxygen species, and bactericidal peptides (13, 14). Inflammatory molecules of haptoglobin (HP), C-Type Lectin Domain Family 4 Member D (CLEC4D), and G-protein coupled receptor 84 (GPR84) are glycoproteins involved in cardiovascular disease and vasculitis (15–17). Therefore, the purpose of this study was to identify the influence of HP, CLEC4D, and GPR84 on leukocytes and their potential clinical prognostic value for KD patients.

## Materials and Methods

### Patients

We enrolled a total of 97 subjects from Kaohsiung Chang Gung Memorial Children's Hospital in Taiwan. Of those, 24 subjects were healthy controls, and 24 subjects were fever controls (with fever but not diagnosed with KD); the other 49 subjects were KD patients who had had blood samples taken both before and after IVIG treatment. All KD patients met the American Heart Association diagnostic criteria for KD (fever of more than 38.0°C ear temperature for at least 5 days, as well as four of the following five symptoms: diffuse mucosal inflammation, bilateral non-purulent conjunctivitis, dysmorphous skin rashes, indurative edematous change over the hands and feet or desquamation over the fingertips or toes, and cervical lymphadenopathy) and were treated with IVIG at the hospital.

All KD patients received a single high dose of IVIG (2 g/kg) over an 8- to 12-h period and were also given aspirin as suggested by the American Heart Association guidelines (18). Patients with any of the following conditions were excluded from this study: symptoms that did not completely match the KD criteria, an acute fever for <5 days, IVIG treatment at another hospital prior to being referred to the study center, treatment with corticosteroids in a form other than the inhaled form within 2 weeks before joining the study, a previous KD diagnosis, or afebrile prior to enrollment. We took blood samples from the KD patients at two points: once before they were treated with IVIG (pre-IVIG) and again at least 3 weeks following IVIG administration (19). We obtained written informed consent from the guardians of all patients aged 0–7 years old or from the patients themselves if they were 7-20 years old using the simpler childhood edition of the informed consent before we collected any samples. The participating children were allowed to withdraw at any time during the study period, and all data were anonymized prior to analysis. The acquisition and subsequent use of samples was approved by the Institutional Review Board of Chang Gung Memorial Hospital, and all of our methods complied with the approved guidelines.

### RNA Isolation and Real-Time Quantitative RT-PCR

To quantify the mRNA levels of genes, we selected the LightCycler® 480 Real-Time PCR System (Roche Molecular Systems, Inc., IN, USA) to perform real-time quantitative PCR. We separated the total mRNA from the WBC using an isolation kit (mirVana™ miRNA Isolation Kit, Catalog number: AM1560, Life Technologies, Carlsbad, CA) and then calculated both the quality (RIN value) and quantity of the RNA samples using Bioanalyzer (ABI) and Qubit (Thermo), respectively, according to the manufacturers' instructions. All RNA samples passed the criterion of RIN≥7. We performed PCR using a SYBR Green PCR Master Mix containing 10 μM of specific forward and reverse primers. We also carried out the relative quantification of gene expression using the comparative threshold cycle (C_T_) method, which enabled us to determine the target amount as 2^−(Δ*CTtarget*−Δ*CTcalibrator*)^ or 2^−ΔΔ*CT*^ (20). Primers were designed to amplify HP, GPR84, and CLEC4D genes and 18S rRNA (internal control) as forward 5′-GTGCCACGCTGATCAATGAA-3′ and reverse 5′-GCAATGTCTTTCGCTGTTGC−3′, 5′-CATGTGGAACAGCTCTGACG-3′ and reverse 5′-CCAGTAGGGTGAGCACATTG−3′, 5′-CCCAGCTGATACCTTCGGTT−3′ and reverse 5′-TGCATGGTGCTCTAACTTGTG−3′, as well as forward 5′-GTAACCCGTTGAACCCCATT-3′ and reverse 5′-CCATCCAATCGGTAGTAGCG-3′, respectively. We performed all experiments twice to verify and validate the amplification efficiencies.

### Statistical Analysis

All data are presented as mean ± standard error. Upon passing the quality control criteria, we evaluated the chips using Partek (Partek, St. Louis), a commercial software specifically designed to analyze microarray data (NCBI GEO: GSE109351). Statistical tests between multiple datasets were analyzed using a one-way analysis of variance (ANOVA) followed by *post hoc* least significant difference (LSD) test or Student's *t*-test as appropriate (3). The receiver operating characteristics curve method using the biological parameters with significance of area under the curve (auROC) was adopted to differentiate between the groups. Correlations between quantitative variables were then assessed using Pearson's coefficient. We carried out all statistical analyses with SPSS version 12.0 for Windows XP (SPSS, Inc., Chicago, USA), and a two-sided *p* < 0.05 was considered statistically significant.

## Results

### HP, CLEC4D, and GPR84 Expressions in the Peripheral White Blood Cells (WBCs) of KD Patients and Controls

**Figure 1:**
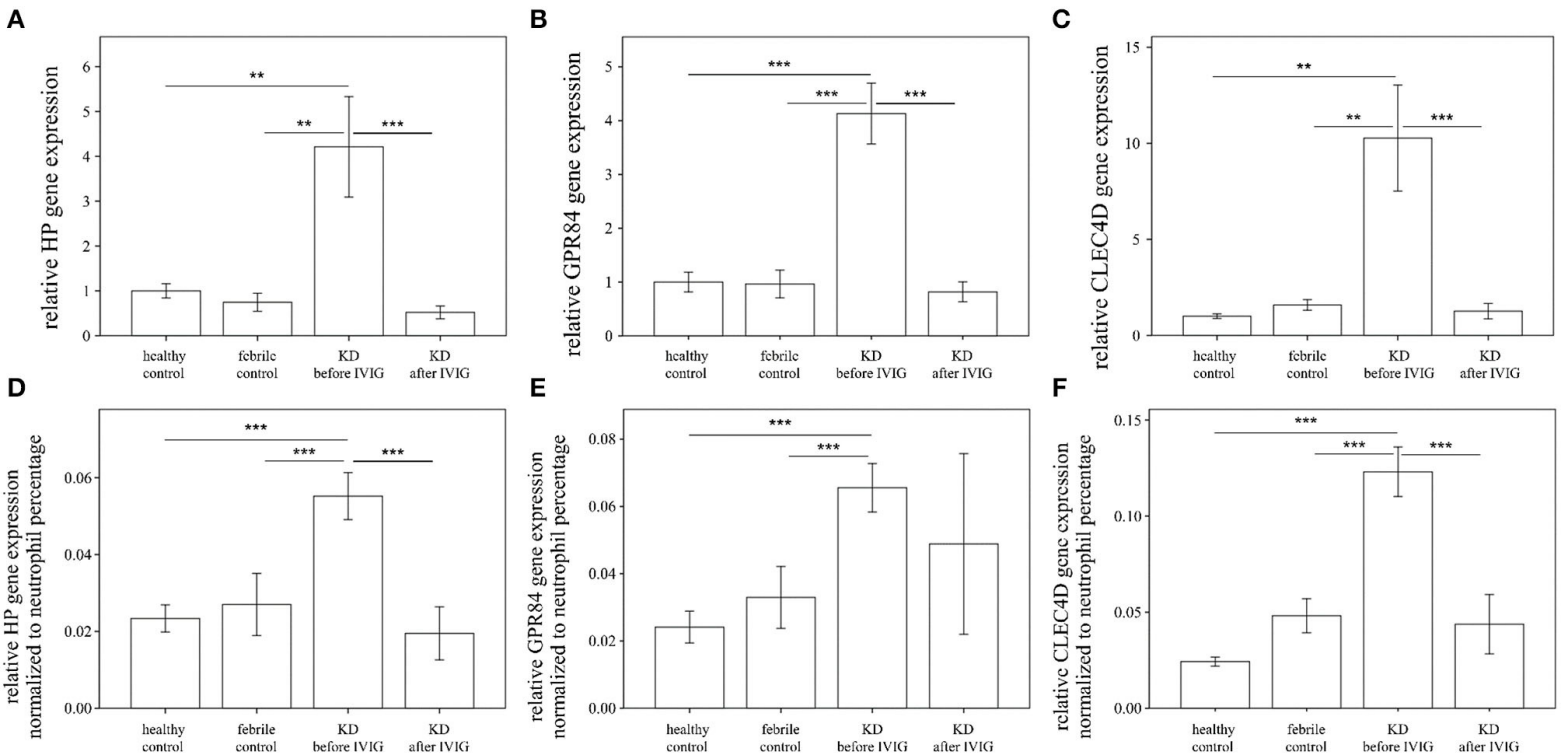
Analyses of HP **(A)**, GPR84 **(B)**, and CLEC4D **(C)** mRNA in the peripheral white blood cells of Kawasaki disease (KD) patients (*n* = 49) and healthy and febrile controls (*n* = 24, respectively) using a real-time quantitative polymerase chain reaction. After normalizing to the neutrophil percentage of each subject, the comparison between cases and controls regarding the expression of HP, GPR84, and CLEC4D are shown as **(D–F)**, respectively, using the analysis of one-way ANOVA followed by *post hoc* least significant difference (LSD) test. Data are expressed as mean ± standard error. IVIG, intravenous immunoglobulin; HP, Haptoglobin; GPR84, G-protein coupled receptor 84; CLEC4D, C-Type Lectin Domain Family 4 Member D; ^**^p < 0.01 and ^***^p < 0.001 between the groups.

In total, this study enrolled 49 KD patients, 24 healthy controls, and 24 febrile subjects (Table 1). We studied the mRNA levels of HP, CLEC4D, and GPR84 genes using real-time quantitative PCR before and after normalizing to the neutrophil percentage of peripheral WBCs in subjects, as shown in Figure 1. HP, GPR84, and CLEC4D gene expressions between KD cases and controls were compared using one-way ANOVA to evaluate the quantitative data and adopting paired samples *t*-test to assess any data changes before and after KD patients underwent IVIG treatment. We then found that the neutrophil percentage of peripheral WBCs demonstrated a positive correlation to each mRNA level of HP (*r* = 468, *p* < 0.001), GPR84 (*r* = 0438, *p* < 0.001), and CLEC4D (*r* = 0.577, *p* < 0.001) upregulation in enrolled subjects by using Pearson's coefficient analysis (Supplementary Figure 1). Compared to both the healthy controls and the fever controls, mRNA levels of HP, CLEC4D, and GPR84 were significantly upregulated in acute-phase KD patients, regardless of whether or not they were normalized to neutrophil percentage. Furthermore, mRNA levels of HP, CLEC4D, and GPR84 genes decreased considerably after KD patients underwent IVIG treatment, compared to before IVIG administration. However, after normalizing to neutrophil percentage, the mRNA level of GPR84 did not show a significant difference between before and after KD patients underwent IVIG treatment.

**Table 1:** Baseline characteristics of patients with KD and controls.

**Characteristic**	**Healthy controls *n* = 24**	**Febrile controls *n* = 24**	**KD *n* = 49 (before IVIG/after IVIG)**	***p*-value (one-way ANOVA)**
Male gender, *n* (%)	14 (58.3)	15 (62.5)	34 (69.4)	0.817
Age (y)	7.5 ± 1.0^a^	2.6 ± 0.3^b^	1.9 ± 0.3^b^	<0.001
Age range (y)	0–16	0–5	0–9	
WBC (1000/uL)	7.9 ± 0.6^a^	8.5 ± 0.7^a^	14.7 ± 0.8^b^/9.7 ± 0.5	<0.001
Neutrophil (%)	43.6 ± 3.1^a^	40.0 ± 3.8^a^	60.0 ± 2.4^b^/33.1 ± 2.0^c^	<0.001
Lymphocyte (%)	46.1 ± 3.1^a^	46.6 ± 3.4^a^	28.7 ± 2.2^b^/56.9 ± 2.0^c^	<0.001
Neutrophil-lymphocyte ratio	1.5 ± 0.5^a^	1.3 ± 0.3^a^	3.5 ± 0.5^b^/0.8 ± 0.2^c^	<0.001
RBC (million/Ul)	4.9 ± 0.1^a^	4.7 ± 0.1^a^	4.2 ± 0.1^b^/4.5 ± 0.1^c^	<0.001
Hemoglobin (g/dL)	12.7 ± 0.2^a^	12.2 ± 0.2^a^	11.0 ± 0.1^b^/11.9 ± 0.2^c^	<0.001
CRP (mg/L)		23.5 ± 4.5^a^	97.9 ± 10.4^b^/1.7 ± 0.4^c^	<0.001
CAL formation			23	
IVIG resistance			9	

CAL, coronary artery lesion; IVIG, intravenous immunoglobulin; KD, Kawasaki disease.

data expressed as mean ± SEM.

a, b, c *Indicates statistical significance between different superscript symbols (p < 0.05) using analysis of one-way ANOVA followed by post hoc least significant difference (LSD) test*.

**Figure 2:**
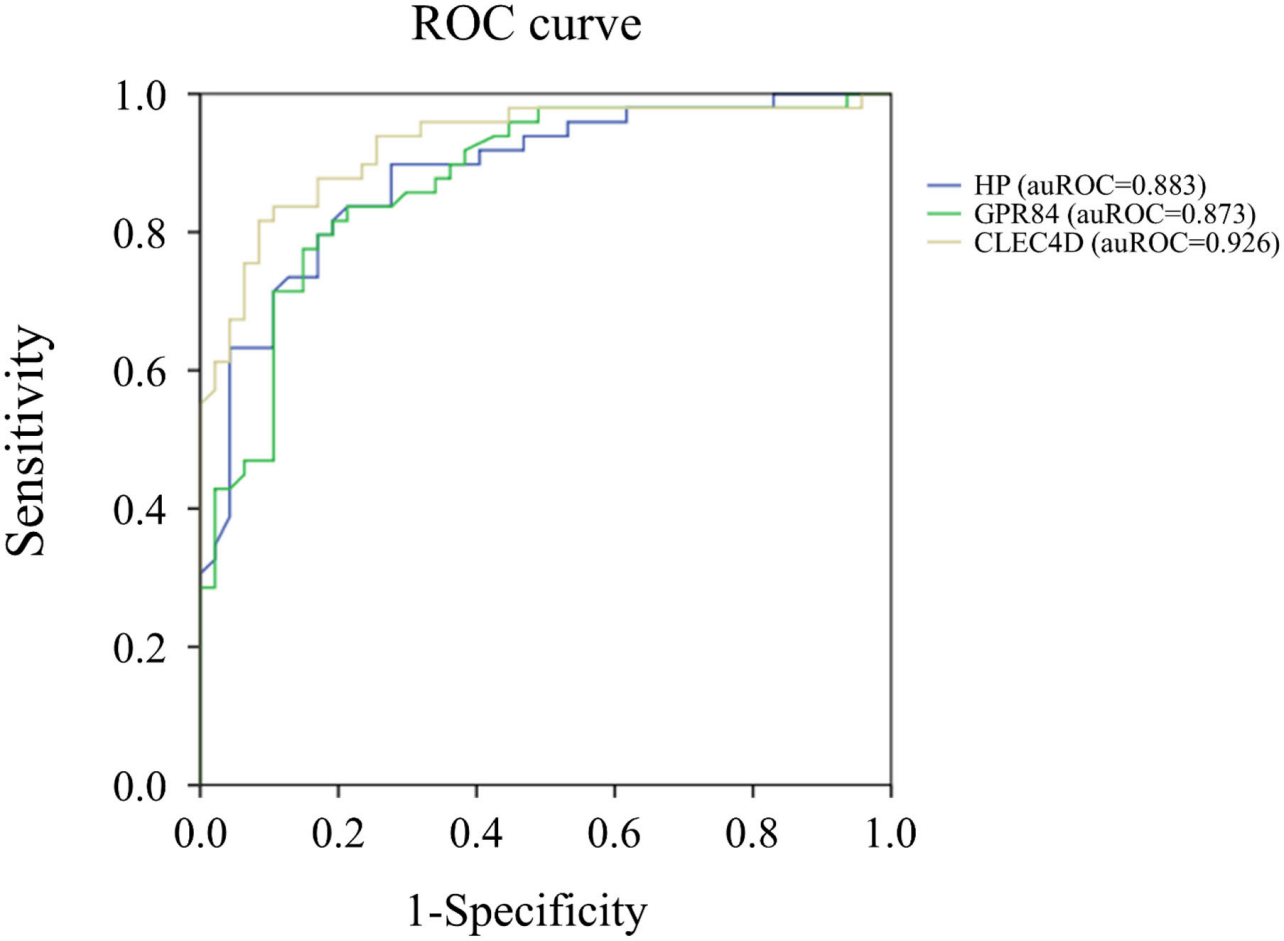
Analyses of the ROC curve to predict Kawasaki disease (KD) using the mRNA levels of HP (blue color), GPR84 (green color), and CLEC4D (gold color) expression. The area under the curve (auROC) of HP, CLEC4D, and GPR84 expression between Kawasaki disease (KD) patients (*n* = 49) and controls (*n* = 48) is 0.883, 0.926, and 0.873, respectively, with regard to predicting KD.

**Figure 3:**
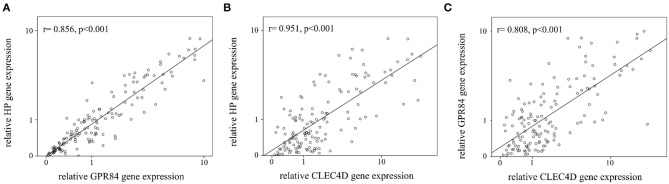
Analyses of the correlation between mRNA levels of HP, GPR84, and CLEC4D in Kawasaki disease (KD, *n* = 49). The positive paired correlation of the relative expression is shown between HP and GPR84 **(A)**; HP and CLEC4D **(B)**; and GPR84 and CLEC4D **(C)**, respectively, using Pearson's coefficient analysis (all, *p* < 0.001).

Notably, the auROC of HP, CLEC4D, and GPR84 expression was 0.883, 0.926, and 0.873, respectively, with regard to predicting KD (Figure 2). Furthermore, the gene expressions of HP, CLEC4D, and GPR84 were highly consistent and demonstrated a positive correlation with each other (Figure 3; all *p* < 0.001).

### Higher HP, CLEC4D, and GPR84 Expressions in the Peripheral White Blood Cells (WBCs) of KD Patients With IVIG Resistance

**Figure 4:**
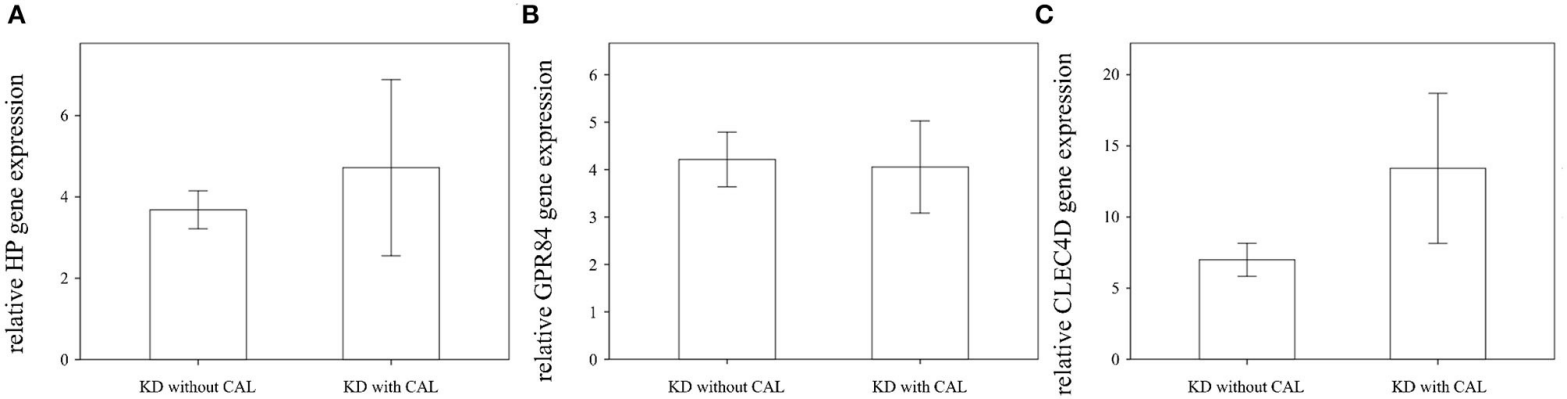
Comparison of relative HP, GPR84, and CLEC4D gene expression in Kawasaki disease (KD) patients with (*n* = 23) or without (*n* = 26) coronary artery lesion (CAL) using Student's *t*-test analysis, shown in **(A–C)**, respectively. The expression of these three genes are not significantly associated with CAL formation in KD patients.

**Figure 5:**
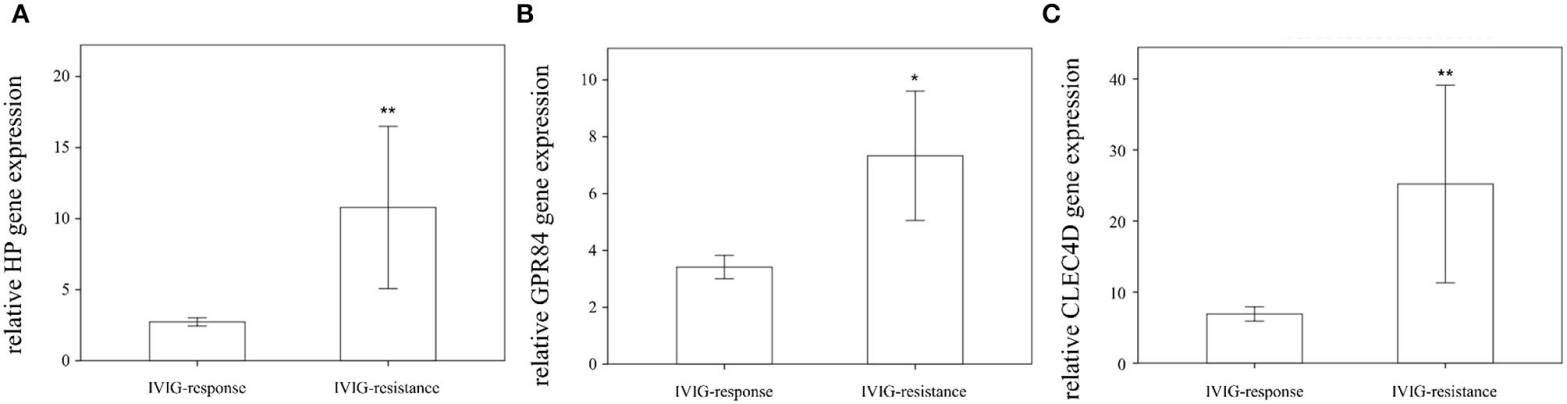
Comparison of relative HP, GPR84, and CLEC4D gene expression in Kawasaki disease (KD) patients regarding IVIG responsiveness (*n* = 40) or IVIG resistance (*n* = 9) using Student's *t*-test analysis, shown in **(A–C)**, respectively. ^*^statistically significant (*p* < 0.05); ^**^statistically significant (*p* < 0.01); IVIG: intravenous immunoglobulin.

We found that the expression of HP, CLEC4D, and GPR84 did not significantly differ between KD patients with and without coronary artery lesion formation (Figure 4). Interestingly, we observed that the mRNA level of HP, CLEC4D, and GPR84 genes were higher in KD patients with resistance to IVIG treatment, as shown in Figure 5, compared with responsiveness to IVIG treatment (*p* = 0.003, 0.006, and 0.024).

## Discussion

In leukocytes, the neutrophils and macrophages play an important role in the innate immune system. They quickly arrive at sites of infection and form the first line of defense against invading micro-organisms via such innate receptors as TLRs, RLRs, and NLRs. They also play a major role in inflammation and tissue injury, contributing to the pathogenesis of various inflammatory diseases, including rheumatoid arthritis, inflammatory bowel disease, and acute respiratory distress syndrome. Bacterial and candida polysaccharides related to the innate immunity responses of the host's glycoprotein contribute to the pathogenesis of inflammatory diseases, including KD. Previous studies about such glycoprotein genes as HP and CLEC4D primarily focused on their expression of KD animal models or human coronary artery tissue (21–23). However, discussion about their expression on the peripheral leukocytes of KD was rare. Moreover, the role of GPR84 up-regulation in KD was firstly discussed in this study. We identified the influence of these glycoprotein genes with regard to leukocyte activation in the pathogenesis of KD. This study revealed higher gene expressions of HP, GPR84, and CLEC4D in KD patients compared to the control subjects. In the multivariable analysis of the ROC curve, HP, CPR84, and CLEC4D mRNA levels had a higher predictive value of KD than other variables, such as WBC and CRP, as shown in Table 1. Furthermore, the upregulation of these three genes was associated with a refractory response to IVIG in KD patients, and a positive correlation was found between their gene expressions.

However, when comparing CAL formation in KD patients, the expressions of the three glycoprotein genes did not differ significantly (Figure 4). CLEC4D (CLECSF8), a characterized member of the “Dectin-2 cluster” of C-type lectin receptors, is expressed by myeloid cells and triggers cellular activation through Syk kinase (24, 25). In animal models of KD (21, 26), the polysaccharides of fungal or bacterial cell walls were capable of inducing murine severe vasculitis via Dectin-1 or 2 recognition. Oharaseki et al. indicated that recognition of alpha-mannan by Dectin-2 played a crucial role in the onset of vasculitis in the studied murine model (21). Therefore, CLEC4D regulation may be involved in the pathogenesis of KD. Furthermore, Rowley et al. demonstrated that the immune transcriptional profile in KD coronary artery tissues had such features as activated cytotoxic T lymphocyte and type I interferon-induced gene upregulation (23). They also indicated that the up-regulation of CLEC4D played a role in the vasculitis of KD patients (27). In this study (Figure 4), the relative up-regulation of CLEC4D mRNA levels regarding CAL formation was greater than others. Moreover, the auROC of CLEC4D mRNA levels was the largest (0.926) of all, and the hyper-expression of CLEC4D was significantly associated with IVIG resistance (*p* < 0.01). Therefore, we supposed that the up-regulation of the CLEC4D gene in leukocytes may play an important role in CAL by inducing a more inflammatory response. However, this hypothesis warrants additional study in the future.

The resting expression of GPR84 is generally low, but it is highly inducible in inflammation (28, 29). In human innate immunity, the G-protein-couple receptor (GPR84) was also up-regulated on both macrophages and neutrophil granulocytes following LPS stimulation (30). GPR84 is primarily expressed in immune-related tissues, such as the spleen, bone marrow, and peripheral blood leukocytes. In the relationship between inflammation and fatty acid sensing or regulation, the ligands for GPR84 play significant roles and may represent a novel drug target for treating immune-mediated diseases and fibrosis, including the experimental neuro-inflammatory model or chronic kidney disease (27, 31, 32). However, to the best of our knowledge, the up-regulation of GPR84 on leukocytes with regard to Kawasaki disease was first noted in this study. Such novel drugs other than IVIG could potentially serve as adjuvant therapy for KD in the future.

Serum HP, an acute-phase protein synthesized primarily from hepatocytes, is known to be involved in coronary artery diseases and Kawasaki disease (22, 33). Serum HP exerts a broad range of anti-inflammatory activities and indirectly acts as a bacteriostatic agent and an antioxidant by binding free hemoglobin (Hb). Kim et al. demonstrated that HP transcription is also found via neutrophils and myeloid cells in response to activation (34). Such neutrophil-derived HP may reduce tissue damage and bacterial growth at infection or injury sites by propagating anti-inflammatory activities and Hb clearance. In line with these findings, this study indicates that HP gene hyper-expression of leukocytes may be associated with higher damage or inflammation of vasculitis, as well as the refractory response to IVIG therapy. Furthermore, HP regulates human iron metabolism by facilitating Hb immediate clearance by macrophages (22, 34). Previous studies have indicated that serum HP elevation or phenotypes are involved in hepcidin-induced iron deficiency anemia and disease outcomes in KD (22, 35). Nevertheless, further research into inflammation-induced HP derived by leukocytes is required to clarify its role in the pathogenesis of KD.

In a systematic meta-analysis review, the neutrophil to lymphocyte ratio (NLR) was shown as an independent risk factor of initial IVIG resistance in KD (36). However, recent studies have indicated that the predictive value of NLR alone was not good enough to determine IVIG resistance and CAL formation in KD (37, 38). We supposed that the neutrophil percentage of peripheral WBCs may play a role in this study. In addition, the percentage of neutrophil demonstrated a positive correlation with the expression of each of HP, GPR84, and CLEC4D in this study. Furthermore, we studied the mRNA levels of HP, CLEC4D, and GPR84 genes using real-time quantitative PCR before and after normalizing to neutrophil percentage. In Figure 1, the mRNA levels of HP, CLEC4D, and GPR84 were significantly upregulated in KD patients, compared with the healthy and febrile controls. Therefore, both NLR and the activation of glycoprotein genes such as HP, GPR84, and CLEC4D may be important biomarkers in the acute phase and IVIG resistance of KD.

## Conclusion

This study is the first to indicate that hyper-expression of the CLEC4D, GPR84, and HP genes with regard to peripheral leukocytes may be appropriate predictors for acute-phase KD, compared with the control subjects. This finding indicates that the activation of glycoprotein genes in leukocytes is important in the acute phase of KD. Furthermore, the up-regulation of all these glycoprotein genes is associated with a refractory response to initial IVIG therapy. It implies that a more inflammation-induced expression of these genes via innate immunity require other anti-inflammatory agents besides IVIG therapy.

## Data Availability Statement

The raw data supporting the conclusions of this article will be made available by the authors, without undue reservation.

## Ethics Statement

The studies involving human participants were reviewed and approved by The Institutional Review Board of Chang Gung Memorial Hospital. Written informed consent to participate in this study was provided by the participants' legal guardian/next of kin.

## Author Contributions

Y-LY, M-HL, and X-YC: data curation. K-CK: formal analysis, writing–original draft, and writing–review and editing. Y-HH: funding acquisition. M-HL and Y-LY: investigation. M-HL, X-YC, and H-CK: methodology. H-CK and Y-HH: project administration, supervision, and validation. X-YC and Y-LY: software. All authors contributed to the article and approved the submitted version.

## Conflict of Interest

The authors declare that the research was conducted in the absence of any commercial or financial relationships that could be construed as a potential conflict of interest.
