# Supplementary figures and images for: The Expression of Glycoprotein Genes in the Inflammatory Process of Kawasaki Disease
Source: Front Pediatr. 2020 Dec 3;8:592122. doi: 10.3389/fped.2020.592122 (PMC7744457; doi:10.3389/fped.2020.592122)

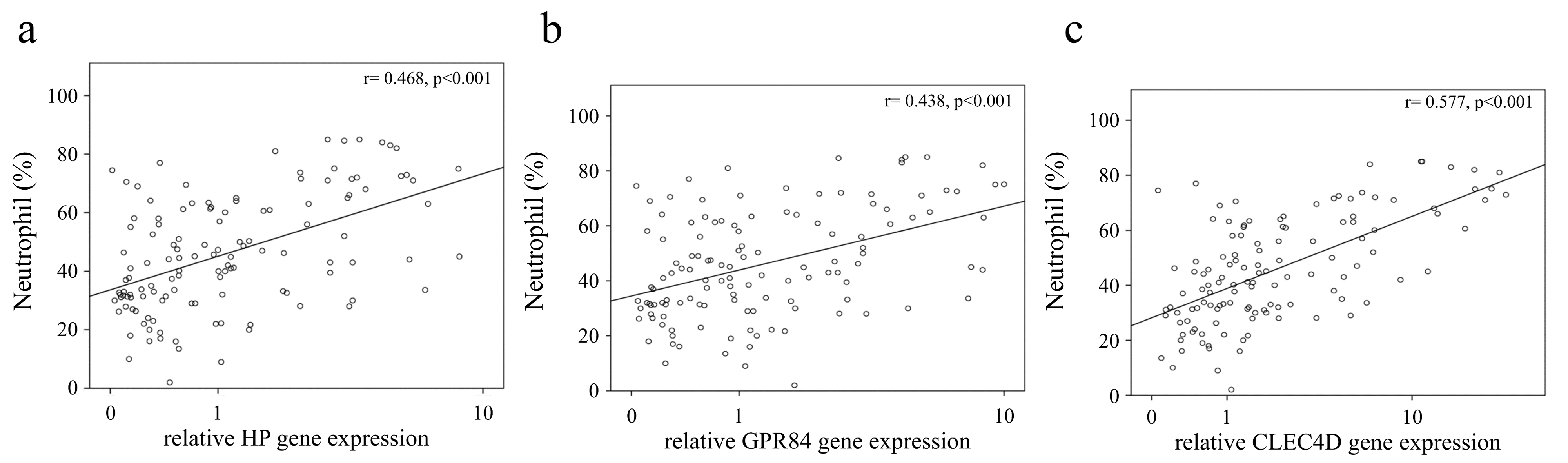

Supplement: Supplementary file 1 [file Image_1.TIF]
